# CAR T Cells Redirected to CD44v6 Control Tumor Growth in Lung and Ovary Adenocarcinoma Bearing Mice

**DOI:** 10.3389/fimmu.2020.00099

**Published:** 2020-02-04

**Authors:** Simona Porcellini, Claudia Asperti, Stefano Corna, Eleonora Cicoria, Veronica Valtolina, Anna Stornaiuolo, Barbara Valentinis, Claudio Bordignon, Catia Traversari

**Affiliations:** Research Department, MolMed SpA, Milan, Italy

**Keywords:** CAR T, CD44v6, adoptive therapy, GMP grade, solid tumor

## Abstract

The main challenge of adoptive therapy with Chimeric Antigen Receptor modified T cells (CAR T) is the application to the field of solid tumors, where the identification of a proper antigen has emerged as one of the major drawbacks to CAR T cell treatment success. CD44 is a glycoprotein involved in cell-cell and cell-matrix interactions. The isoform containing the variant domain 6 of CD44 gene (CD44v6) has been implicated in tumorigenesis, tumor cell invasion and metastasis and represents an attractive target for CAR T cell therapies. Targeting CD44v6 antigen has been shown to control tumor growth in acute myeloid leukemia and multiple myeloma mouse models. While CAR T approach for the treatment of B cell malignancies has shown great success, response rates among patients with solid cancer are less favorable. The purpose of our study was to test the efficacy of CD44v6.CAR T cells, produced in compliance with Good Manufacturing Practice (GMP), in adenocarcinoma tumor models. We generated a bicistronic retroviral vector containing the CD44v6 CAR and the HSV-TK Mut2 suicide gene to enhance the safety of the proposed CAR T cell therapy. CD44v6 transduced CAR T cells were homogeneously positive for ΔLNGFR selection marker, were enriched in T central memory (T_CM_) and T memory stem cells (T_SCM_) and displayed a highly activated phenotype. *In vitro* assays revealed antigen-specific activation and cytotoxicity of human CD44v6.CAR T cells against CD44v6 expressing tumor cell lines. When infused in immunodeficient tumor bearing mice, human CD44v6.CAR T cells were able to reach, infiltrate and proliferate at tumor sites, finally resulting in tumor growth control. Next, we checked if cells produced in compliance with GMP grade standards retained the same antitumor activity of those produced with research grade materials and protocols. Noteworthy, no differences in the potency of the CAR T obtained with the two manufacturing processes were observed. In conclusion, our preclinical results suggest that CD44v6.CAR T based adoptive therapy could be a promising strategy in solid cancer treatment.

## Introduction

Adoptive cell therapy with CAR redirected T cells is considered one of the most promising option in the field of personalized cancer treatments. Currently, the CD19.CAR T cell therapy Novartis's Kymriah and Gilead's Yescarta, have been approved for the treatment of adult relapsed or refractory lymphomas and pediatric relapsed acute lymphoblastic leukemias based on the striking clinical results obtained in global phase II trials, where high and durable remission rates were obtained (1, 2). However, the impressive clinical responses achieved in the treatment of hematological malignancies were not observed in solid tumors, because of the difficulties of T cells to infiltrate and survive into the tumor microenvironment (3, 4) as well as by the paucity of tumor-specific target antigens with expression patterns broadly expressed across different tumor histotypes (5).

CD44 is a glycoprotein involved in cell-cell and cell-matrix interactions and its structural heterogeneity is mainly caused by alternative splicing (6). CD44 variant isoforms, in particular those containing CD44 variant domain 6 (CD44v6), have been implicated in tumorigenesis, tumor cell invasion and metastasis (7). CD44v6 is expressed in hematological tumors such as acute myeloid leukemia (AML) and multiple myeloma (MM) cells. Noteworthy, it has been reported that CD44v6-silenced AML and MM cells are severely impaired in their capacity to engraft in immunocompromised mice (8), thus providing a rationale for targeting the CD44v6 antigen on AML and MM, because the potential generation of antigen lost variants is overcome by the reduced tumor growth of CD44v6-negative tumor cells. Concerning the expression on normal hematopoietic tissues, CD44v6 is absent on hematopoietic stem cells, progenitors, and resting T and B lymphocytes, but it is expressed by circulating monocytes (8).

CD44v6 plays an important role also in solid tumor growth and metastasis development. Its expression has been extensively studied in several tumor types, and detected in breast, colon, gastrointestinal, bladder, ovarian, and lung cancers (9). In particular, all squamous cell lung carcinomas, 45% of lung adenocarcinomas (10), and 66.1% of ovarian cancers (11) analyzed for the expression of CD44v6, were found positive. On normal epithelial tissues, detectable CD44v6 expression has been reported only in skin and oral mucosa, albeit at considerably lower levels than in primary leukemic blasts (8). Low-level CD44v6 expression was, indeed, confirmed on primary cultured keratinocytes (8) that, however, were not killed by CD44v6.CAR T cells, at effector to target ratios allowing potent antitumor effect (8), thus supporting a reasonable therapeutic index for the clinical application of the CD44v6.CAR T.

Recently, preclinical studies on safety and efficacy of a bicistronic retroviral vector encoding a new CAR specific for the target antigen CD44v6 and the HSV-TK Mut2 suicide gene (12) have been reported in the hematological setting (8, 13). Moreover, an upcoming first-in-man Phase I/II clinical trial (clinical trial.gov NCT04097301) is currently on going to assess the safety, efficacy and feasibility of CD44v6.CAR T cell immunotherapy in acute myeloid leukemia (AML) and multiple myeloma (MM) patients.

Purpose of this study is to investigate the activity of CD44v6.CAR T cells in *in vitro* and *in vivo* human models of lung and ovary adenocarcinomas. We first showed that CD44v6.CAR T cells are functionally activated *in vitro* and have the capacity to infiltrate, proliferate and inhibit tumor growth *in vivo*, enhancing overall survival of treated mice. In addition, we characterized CD44v6.CAR T cells manufactured complying with GMP-grade process, demonstrating that they preserve immunophenotype and functional activities comparable to those of CAR T cells produced with research grade processes.

## Materials and Methods

### Retroviral Vector Design and Production

An LXSN-based retroviral vector (14) (Genebank accession # 28248), carrying the HSV-TK Mut2 suicide gene (12) and the ΔLNGFR marker gene, was used to generate the CD44v6 CAR retroviral vector. This vector carried the HSV-TK Mut2 gene under the transcriptional control of the viral 5'LTR and an internal expression cassette with the SV40 promoter driving the CD44v6 CAR. The CD44v6 CAR includes the single chain variable fragment (scFv) of the humanized mAb BIWA-8 (15), specific for the CD44v6 antigen, a spacer formed by the extracellular domain of the human low-affinity nerve growth factor receptor (ΔLNGFr), that allows recognition and selection of CAR transduced cells, the transmembrane and intracellular domain of the human costimulatory molecule CD28, and the intracellular signaling domain of the human CD3ζ chain. The same cloning strategy was applied to generate the control vector encoding a CAR specific for the CD19 antigen (CD19 CAR) (13) and the HSV-TK Mut2 suicide gene. In some experiments as control we used the SFCMM3 Mut2 retroviral vector, where the CAR was substituted by the extracellular domain of the ΔLNGFr (12).

### Generation of CD44v6 and CD19 CAR T Cells

Peripheral blood mononuclear cells (PBMCs) were isolated from buffy coats or leukapheresis of healthy donors (San Raffaele Hospital; AllCells) by density gradient centrifugation (Lymphoprep, Fresenius). PBMCs were cultured in Xvivo 15 w/o gentamicin and phenol red (Lonza) supplemented with 3% AB human plasma (Kendrion), 100 U/ml Penicillin and Streptomycin (Lonza), 2 mM L-Glutamine (Lonza), 100 U/ml MACS GMP Recombinant IL7 and 200 U/ml MACS GMP Recombinant IL15 (Miltenyi Biotec). PBMCs were activated with CD3/CD28 magnetic beads (Dynal, Invitrogen) or MACS® GMP T Cell TransAct (TransAct; Miltenyi Biotec). Activated cells were transduced with retroviral vectors in the presence of Retronectin (Takara). Cells were than cultured and expanded before selection for ΔLNGFr expression with CliniMACS CD271-LS Reagent (Miltenyi Biotec). For both protocol, ΔLNGFr positive cells were frozen 10 days after stimulation. At the day of mice treatment, CAR T cells where thawed in presence of 10 U/ml Benzonase, cultured for 4 h in presence of 1 U/ml Benzonase (MerckMillipore), then counted and infused.

### Target Cell Lines

The following cell lines of epithelial origin (EpCAM^+^ cells) were analyzed for CD44v6 expression: MR232 lung adenocarcinoma (16), A-427 (ATCC) lung carcinomas, IGROV-1 (NCI tumor repository), and SKOV-3 ovarian adenocarcinoma (ATCC), JU77 mesothelioma (CellBank Australia), HCT116 (ATCC) and T84 colon carcinoma (ATCC), BxPC-3 pancreatic cancer (ATCC) and PC-3 prostatic cancer (17). The following cell lines were used as CD44v6^−^ control cells: MOLT-4 lymphoblastic acute leukemia (ATCC), or CD19^+^ control, BV-173 B cell precursor leukemia (18).

### Flow Cytometry and TCRVβ Repertoire Analysis

T cells were stained with a panel of antibodies including monoclonal mouse anti-human CD271, CD3, CD45, CD4, CD8, CD45RA, CD62L, HLA DR, PD1, CD25 (BD Biosciences). Tumor cells were stained with anti-CD44v6 and anti-EpCAM (BD Biosciences), and the expression level reported as RFI (Relative Fluorescence Intensity). Murine cells were stained with Rat anti-mouse H-2K^d^ (Bio Legend), and anti-CD45 (BD Biosciences). Viability Stain 510 (BD Bioscience) was used to detect live cells. TCR Vβ Repertoire analysis was performed with IOTest® Beta Mark Kit (Thermo Fisher Scientific). T cell proliferation was evaluated with mouse anti-Ki-67 Set (BD Biosciences). All samples were acquired with a (FACSCanto™ system, BD Biosciences) and analyzed using DIVA software.

### *In vitro* Functional Assays

Degranulation, measured by cell surface modulation of CD107a (19), and intracellular cytokines production (TNF-α, IFN-γ, IL-2), were analyzed by flow cytometry in CAR T cells incubated with different target cells or left alone. Briefly, CD44v6.CAR T and CD19.CAR T cells from different donors, at day 11–15 after stimulation with CD3/CD28 beads, were left untreated or stimulated with target cells at the ratio of 1:1. Anti CD107a Ab (Miltenyi), Monensin and Brefeldin (BD Biosciences) were added during the incubation period. As positive control, CAR T cells were stimulated with 10 ng/ml phorbol myristate acetate (PMA; Sigma), and 1 μg/ml Ionomycin (IONO; Sigma). After 5 h of incubation, cells were stained with anti CD3 Ab (BD Bioscience) and Viability Stain 510 (BD Bioscience), fixed, permeabilized (Cytofix/Cytoperm kit, following manufacturer's instruction; BD Bioscience), and then stained for intracellular cytokines with TNF-α (BD Bioscience), IFN-γ (BD Bioscience), and IL-2 (BD Bioscience) specific Abs. Cells were subjected to flow cytometry and viable, CD3^+^ cells analyzed for TNF-α, IFN-γ, IL-2, or CD107a expression. The percentage of positive CAR T cells left alone was subtracted to the percentage of positive CAR T cells stimulated with the different targets or PMA/IONO. For bioluminescence *in vitro* killing assay, CD44v6 and CD19.CAR T cells were co-cultured with luciferase-expressing tumor cells at various effector to target cells ratio (1:10-1:5-1:1) in flat transparent bottom black 96-well plates. Co-cultures were analyzed for luminescence 48–72 h later using Caliper IVIS Spectrum. For antigen stimulation and proliferation assays, CD44v6 and CD19.CAR T cells were co-cultured with irradiated confluent target cells, at a concentration of 10^6^ CAR^+^ T cells per ml in 24-well tissue culture plates. Identical stimulations in fresh medium were performed three times under the same conditions. Total cells were counted and analyzed weekly by flow cytometry.

### *In vivo* Xenograft Models

Experimental protocols were approved by the Institutional Animal Care and Use Committee of San Raffaele Scientific Institute (IACUC 725). NOD.Cg-*Prkdcscid Il2rgtm1Wjl*/SzJ (NSG) transgenic mice of 8 weeks of age were provided by Charles River Laboratories. Mice were injected subcutaneously with 0.3 × 10^6^ IGROV-1 tumor cells, except for experiment shown in **Figure 3** where mice were inoculated with 7 × 10^6^ tumor cells. At day 7 mice received, via tail vein injection, CD19 or CD44v6.CAR T cells. From five to eight mice per group were used. Tumors were measured by caliper and tumor volume was calculated using the equation l^2^ × L where l is the shortest diameter and L is the longest. Animal Bioluminescence imaging (BLI) was performed by using the IVIS SpectrumCT System (Perkin Elmer, USA). Each mouse received an intra-peritoneal injection of 150 mg luciferin/kg body weight 10 min before BLI. Animals were kept at 37°C and under gaseous anesthesia. A set of images every 2 min from 10 to 20 min were acquired, after luciferin injection, in order to detect the highest BLI signal. Images were analyzed by measuring the total flux (photons/seconds) within the ROI (Software Living Image 4.5, Perkin Elmer). For MR232 tumor model, mice were injected subcutaneously with 0.3 × 10^6^ MR232 tumor cells. At day 3, mice received via tail vein injection CD19 or CD44v6.CAR T cells. From five to eight mice per group were used. At different time points, tumors were harvested and embedded in OCT for immunofluorescence or digested by a mixture of collagenase and dispase for 1 h. Single cell suspensions were immediately stained or plated in culture for further analysis (immunophenotype, TCR Vβ Repertoire analysis, CD44v6 expression, luciferase-based killing assays).

### Immunohistochemistry and Fluorescence Imaging

Cryostat sections (10 μm thickness) of OCT embedded tumors, were dried on Superfrost Plus slides (Fisher Scientific), fixed with 4% paraformaldehyde and then incubated with 0.2% Triton X-100, 10% normal goat serum to block non-specific antibody binding. Sections were incubated for 15–18 h with primary antibodies at 4°C. Rabbit monoclonal anti-CD3 (AbCam) and rabbit monoclonal anti-CK7 (AbCam) were used. Secondary antibodies were Goat anti rabbit Alexa568 and Alexa488 (Invitrogen), respectively. Tissue sections were examined with an Olympus BX61 fluorescence microscope equipped with single, dual and triple fluorescence filters, and a Colorview camera (Olympus). Digital images of tumors in sequential cryostat sections of each tumor were captured.

### Statistical Analysis

Data comparison was performed by using 1-tailed Student *t*-test or ANOVA test. Bonferroni correction was applied for multiple comparison. Differences with a *P* < 0.05 were considered statistically significant. To determine the overall survival of CD44v6 treated mice, Kaplan-Meier analyses was performed and the log-rank Mantel-Cox test was employed to determine any statistical difference between the survival curves of the cohorts.

## Results

### T Lymphocytes Expressing the CD44v6-Specific CAR Are Activated and Displayed Cytotoxic Activity Against CD44v6^+^ Tumor Cell Lines

**Figure 1:**
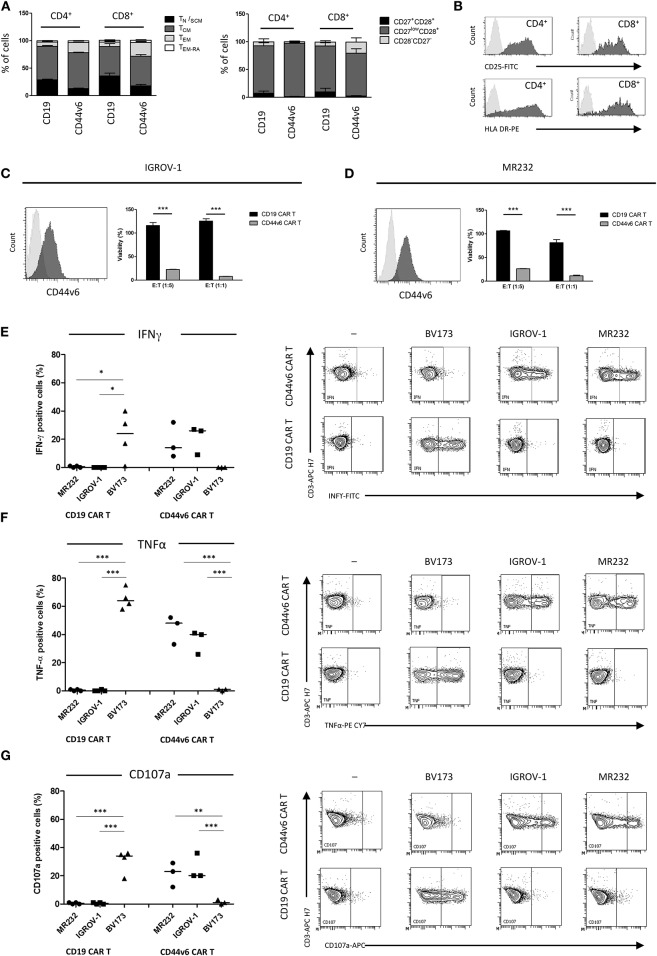
Composition and *in vitro* activity of CD44v6.CAR T cell product adoptively transferred into mice. T cell memory phenotypic analysis based on CD45RA and CD62L (**A**, left panel) or CD27 and CD28 (**A**, right panel) expression. T_N/SCM_ (CD45RA^+^/CD62L^+^ memory stem T cells), T_CM_ (CD45RA^−^/CD62L^+^ central memory T cells), T_EM_ (CD45RA^−^/CD62L^−^ effector memory T cells), T_EM−RA_ (CD45^+^/CD62L^−^ effector memory RA T cells). Data, collected at the infusion, are the mean ± SE obtained from three independent experiments. Representative flow cytometry plot demonstrating activation markers expression in CD44v6 transduced cells. Data shown are representative of three independent experiments **(B)**. Cytotoxic activity of CAR T cells against IGROV-1 **(C)** and MR232 **(D)** cell lines. CD19 and CD44v6.CAR T cells were co-cultured for 48 h with luciferase expressing tumor cells at 1:5 and 1:1 E:T ratio. Percentage of viability was calculated measuring residual luciferase activity of the co-cultured cells respect to the target alone. The graphs show mean ± SE of a representative experiment (*n* = 3). Cell surface CD44v6 expression of target cells is shown on the left of each panel. Intracellular TNF-α and IFN-γ production (**E,F**, respectively), and cell surface expression of CD107a **(G)** were analyzed in CD19.CAR T or CD44v6.CAR T cells incubated with the indicated cell lines MR232 and IGROV-1 (CD44v6^+^ and CD19^−^) or BV173 (CD44v6^−^ and CD19^+^), at E:T ratio 1:1, for 5 h. Percentage of positive cells on gated viable CD3^+^ cells from different donors, is shown. *P < 0.05, **P < 0.01, ***P < 0.001 (Bonferroni's Multiple Comparison Test).

Lymphocytes from three healthy donors were engineered to express CD44v6.CAR using a γ retroviral vector (Supplementary Figure 1A). The same retroviral vector carrying CD19.CAR was used as control (Supplementary Figure 1B). After transduction, a mean of 38% (range 34–42%) of the cells expressed CD44v6.CAR as evaluated by FACS analysis (data not shown). After the selection step, 91% (range 84–92%) of T cells were ΔLNGFR^+^. Both CD4 and CD8 T cells stably expressed the CAR, with the CD8^+^ cells expressing the CAR at lower level than CD4^+^ cells (MFI 5011 vs. MFI 7977) (Table 1). In the final product more than 93% of the cells were CD3^+^, with a prevalence of CD4^+^ cells over CD8^+^ cells in two out of three preparations (Table 1). Moreover, CAR T cells were enriched in central memory T cells as determined by CD45RA and CD62L (Figure 1A, left panel), and CD27 and CD28 (Figure 1A, right panel) expression. CD44v6.CAR T cells displayed a highly activated phenotype, with all the cells expressing CD25 and HLA-DR (Figure 1B).

**Table 1:** Immunophenotypic characterization of CAR T cells.

**PBMC ID**		**CD3^+^** **(%)**	**CD3^+^/CD4^+^** **(%)**	**CD3^+^/CD8^+^** **(%)**	**CD3^+^ ΔLNGFR^+^** **(%)**	**CD4^+^ ΔLNGFR^+^** **(MFI)**	**CD8^+^ ΔLNGFR^+^** **(MFI)**
#79	CD19ΔN	99	78	17	91	7,113	4,086
	CD44v6ΔN	100	80	18	92	5,403	3,530
#80	CD19ΔN	99	35	56	79	9,239	6,001
	CD44v6ΔN	98	38	54	84	10,551	6,559
#84	CD19ΔN	99	69	29	83	n.a.	n.a.
	CD44v6ΔN	93	83	14	96	n.a.	n.a.

To identify the target cell lines for CD44v6 redirected CAR T cell studies, we analyzed by FACS a panel of tumor cell lines of epithelial origin including mesothelioma (JU77), ovarian (SKOV-3, IGROV-1), lung (A427, MR232), colon (T84, HCT116), prostatic (PC-3) carcinomas, and pancreatic adenocarcinomas (BxPC-3) (Supplementary Figure 2). CD44v6 was expressed at variable level independently of the tumor histotype. Its expression was low or undetectable (RFI ≤ 1.5) in the A427 lung and PC-3 prostatic cell lines, whereas CD44v6 was detected at high level in one colon carcinoma (HCT116) and in the pancreatic BxPC-3 adenocarcinoma cell lines. In the other cell lines, CD44v6 expression was moderate. We focused our study on the ovarian IGROV-1 and the lung MR232 cell lines, which had a moderate CD44v6 expression level (Supplementary Figure 1 and Figures 1C,D) and rapidly engraft in NSG immunodeficient mice (unpublished results).

To evaluate the antigen-specific activation of CD44v6.CAR T cells, we assessed the cytotoxic activity against the two selected cell lines at various E:T ratio, in a 48-h co-culture assay (Figures 1C,D). CD44v6.CAR T, but not control CD19.CAR T cells, significantly killed the CD44v6^+^ targets (22.5 ± 0.5% of residual viability at 1:5 ratio, *P* < 0.001 for IGROV-1, 26.2 ± 0.3% of residual viability at 1:5 ratio, *P* < 0.001 for MR232). The effect was more evident at 1:1 ratio (8.00 ± 0.2% of residual viability *P* < 0.001 for IGROV-1, 11.3 ± 1.4% of residual viability, *P* < 0.001 for MR232). To further demonstrate the killing specificity of CD44v6.CAR T cells, cytotoxic activity was also tested on MOLT-4 and CD44v6-transduced MOLT-4 T lymphoblasts (Supplementary Figure 3). Only CD44v6-transduced MOLT-4 were recognized and killed by CD44v6.CAR T cells (Supplementary Figure 3, right panel). Thereafter, we measured degranulation and cytokine production by CAR T cells co-cultured with IGROV-1 and MR232 target cells (CD44v6^+^ and CD19^−^) or with BV173 cells (CD44v6^−^ and CD19^+^) as control (Figures 1E–G). CD44v6.CAR T cells were specifically and significantly activated only by cell lines expressing the CD44v6 antigen, as demonstrated by TNF-α and IFN-γ production and by degranulation. As expected, CD19.CAR T were not activated by the CD44v6^+^ cell lines, whereas they produced cytokine and degranulated in the presence of CD19^+^ target cells. Both CD44v6 and CD19.CAR T, showed a similar, CAR-independent functional activity when stimulated by PMA/IONO (Supplementary Figure 4).

### T Lymphocytes Expressing the CD44v6-Specific CAR Maintain Cytotoxic Activity Against CD44v6^+^ Tumor Cell Lines After Multiple Stimulation

It is known that repeated antigen stimulation may affect T cell functions and that T cell exhaustion may occur during *ex vivo* CAR T cell expansion because of CAR-mediated tonic signaling (20).

To investigate the ability of CD44v6.CAR T cells to resist exhaustion induced by repeated antigen encounter, we assessed CD44v6 and control CAR T cells for proliferation, cytolytic function, and cytokine production upon weekly stimulation with CD44v6^+^ tumor cells (i.e., IGROV-1).

**Figure 2:**
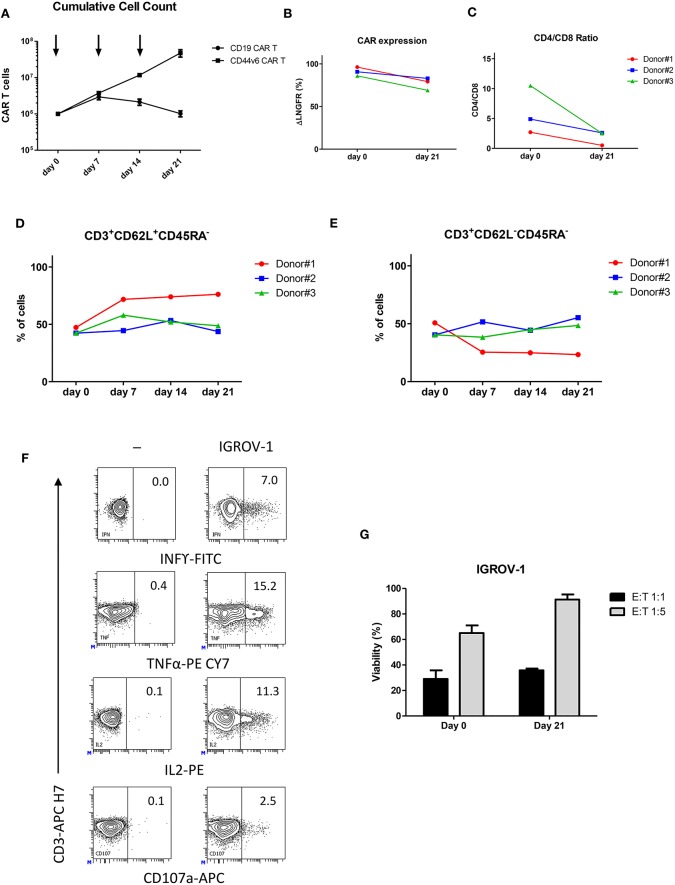
Upon repeated antigen stimulation, CD44v6.CAR T cells proliferate, maintain an indifferentiated phenotype and retain effector cytokine secretion and cytotoxicity *in vitro*. CD19 and CD44v6.CAR T cells were exposed to CD44v6^+^ irradiated cells (IGROV-1) every 7 days for a total of three stimulations. Graph shows cumulative cell counts, mean ± SE (*n* = 3 Donors) **(A)**. CAR.CD44v6 expression at cell surface (ΔLNGFR^+^ cells) **(B)**, CD4/CD8 ratio **(C)**, T cell memory phenotypic analysis based on CD45RA and CD62L, T_CM_ (CD45RA^−^/CD62L^+^ central memory T cells) **(D)**. T_EM_ (CD45RA^−^/CD62L^−^ effector memory T cells) **(E)** at day 0 and at day 21 upon weekly stimulation. After the third antigen stimulation CD44v6.CAR T cells functionality was assessed against IGROV-1 cell line *in vitro*. Intracellular TNF-α, IFN-γ and IL2 production, and cell surface expression of CD107a **(F)** were analyzed in CD44v6.CAR T cells incubated with IGROV-1 (CD44v6^+^) at E:T ratio 1:1, for 5 h. CD44v6.CAR T cells were co-cultured for 72 h with luciferase expressing tumor cells at 1:1 and 1:5 E:T ratio, before and after repeated stimuli. Percentage of viability was calculated measuring residual luciferase activity of the co-cultured cells respect to the target alone. The graph shows mean ± SE of a representative experiment **(G)**.

CD44v6.CAR T cells showed a great T cell expansion rate (21) upon weekly antigen stimulation, maintaining the ability to expand also after the third stimulation (Figure 2A). As expected, the control CD19.CAR T cells lost the ability to expand following the first stimulation (Figure 2A).

During the serial stimulations, expression of CD44v6.CAR on the cell surface (measured as ΔLNGFR marker expression), remained high and comparable to the ΔLNGFR expression at day 0 (Figure 2B). We further analyzed the phenotype of CD44v6.CAR T cells focusing on their relative CD4/CD8 ratio, memory phenotype and expression of exhaustion markers. All donors showed an inversion of the CD4/CD8 ratio, after the third stimulation, consistent with a higher proliferation rate and/or better persistence of CD8 CAR T cells (Figure 2C). Moreover, persisting CD44v6.CAR T cells maintained the same memory phenotype, evaluated as CD62L^+^CD45RA^−^ (central memory) and CD62L^−^CD45RA^−^ (effector memory) expression, throughout the serial stimulations (Figures 2D,E). The expression of the inhibitory receptors PD-1, LAG-3, and TIM-3 showed a slight induction of PD-1 which correlates with the activated status of the cells. No differences in LAG-3 and TIM-3 expression were observed (data not shown). Finally, we assessed the functional activity of the CD44v6.CAR T cells after repeated antigen stimulations. As shown in Figure 2F, CD44v6.CAR T cells retained the capacity to produce cytokines and to degranulate upon antigen stimulation. Of note, in a prolonged cytotoxic test (72 h), CD44v6.CAR T cells were still able to kill target cells at different effector to target ratios, suggesting that CD44v6.CAR T cells had a potent cytotoxic potential even after repeated antigen stimulations (figure 2G).

All together the results are promising for further cell antigenic re-stimulation of CAR T cells *in vivo*.

### CAR-T Cells Were Detected *in vivo* at the Tumor Site

**Figure 3:**
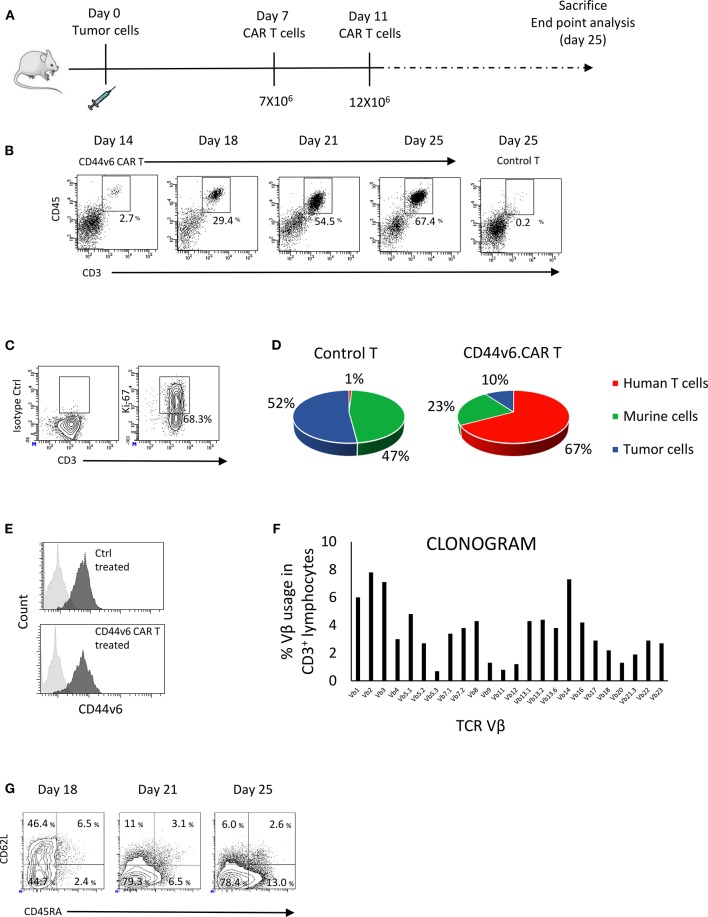
CD44v6.CAR T cells, infused in mice bearing human ovarian carcinoma, infiltrate and proliferate at tumor site. NSG mice were subcutaneously injected with CD44v6^+^ IGROV-1 tumor cells. Seven days later, mice were infused via tail vein injection with 7 × 10^6^ CD19 or CD44v6.CAR T cells and re-infused 4 days later with 12 × 10^6^ CD19 or CD44v6.CAR T cells **(A)**. Mice were sacrificed at different time points and tumors were excised and analyzed by cytofluorimetric analysis. A progressive infiltration of CD44v6.CAR T cells in tumors was evident compared to control **(B)**. CD44v6.CAR T cells isolated from tumors were still proliferating 53 days after infusion (*n* = 3) **(C)**. Quantification of the percentage of CD19 or CD44v6.CAR T and tumor cells after cytofluorimetric analysis **(D)**. Tumor cells isolated by tumors excised from Ctrl group (*n* = 4) and from CD44v6 group (*n* = 4) were CD44v6^+^ **(E)**. Infiltrating T cells displayed a polyclonal TCR Vβ repertoire **(F)**. At later time points, TILs lost CD62L and showed higher CD45RA expression compatible with a differentiated phenotype **(G)**.

CAR T cells may encounter some difficulty in trafficking into solid tumors due to the presence of physical barriers and high interstitial fluid pressure (22). In order to verify if CD44v6.CAR T cells are able to infiltrate solid tumors, we engrafted NOD/SCID/IL2Rnull (NSG) mice subcutaneously with 7 × 10^6^ (high tumor burden setting) IGROV-1 tumor cells. Seven days later mice were infused intravenously (iv) with 7 × 10^6^ CD44v6.CAR T cells and 4 days later with an additional dose of 12 × 10^6^ CD44v6.CAR T cells. Fourteen, eighteen, twenty one and twenty five days after tumor challenge, tumors were harvested, digested, processed into single cell suspension and analyzed for the presence of human CAR T cells (Figure 3A). CD44v6.CAR T cells, identified by human CD45 and CD3 expression, were found at tumor sites in all the mice examined (Figure 3B).

The percentage and the number of infiltrating CAR T cells increased over time, whereas no evidence of control T cells were found at tumor sites (Figure 3B). At sacrifice (day 53 after infusion) more than 68% of the CAR T cells were proliferating as demonstrated by the Ki67 positivity (Figure 3C), suggesting *in situ* proliferation instead of intake from body reservoirs. As shown in Figure 3D, 25 days after engraftment in mice injected with CD44v6.CAR T cells, the 67.4% of the cells were human T cells, whereas in mice injected with control T cells, no human T cells were detected. In agreement, the percentage of tumor cells was greatly reduced in CD44v6.CAR T treated mice. Of note, residual tumor cells from CD44v6.CAR T treated animals expressed CD44v6 at the same level of cells from control treated animals (RFI 7.0 ± 0.3 for control treated group, *n* = 4 and 7.3 ± 0.9 for CD44v6.CAR T treated group, *n* = 4), thus supporting the absence of CD44v6 loss variants selection under CAR T pressure (Figure 3E).

The increase in human T cells over time was not due to a monoclonal T cell proliferation, as demonstrated by flow cytometry Vβ repertoire analysis of infiltrating CD44v6.CAR T cells at day 25 (Figure 3F). Interestingly, during the time point analysis, we observed progressive loss of CD62L and acquisition of CD45RA expression, suggesting an antigen dependent local differentiation of CAR T cells into effector cells (Figure 3G). Altogether, these results demonstrate the ability of CD44v6.CAR T cells to infiltrate and proliferate even in a high tumor burden model, in which tumor rapidly reaches humane endpoints to which mice are euthanized.

### CD44v6.CAR-T Cells Have *in vivo* Antitumor Activity

**Figure 4:**
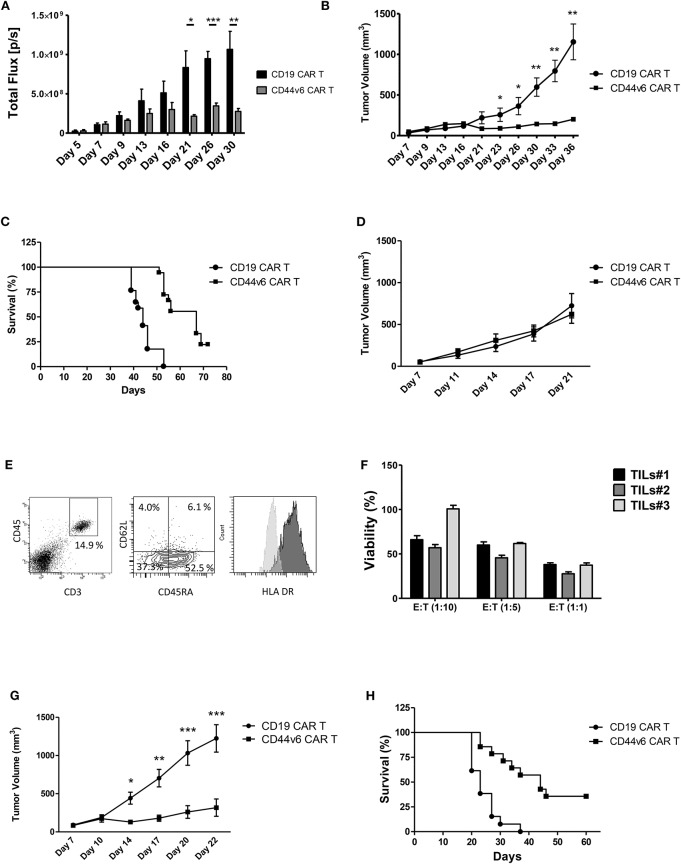
Systemic treatment of mice bearing human ovarian and lung carcinoma with CD44v6.CAR T cells leads to tumor growth control and enhances survival. NSG mice were subcutaneously injected with 3 × 10^5^ CD44v6^+^ IGROV-1 tumor cells, which stably express Firefly Luciferase. Seven days later, mice were infused via tail vein injection with 4.5 × 10^6^ CD19 or CD44v6.CAR T cells and tumor growth quantified by *in vivo* imaging of luciferase expressing tumor cells **(A)** and by measuring tumor size **(B)**. Mice treated with CD44v6.CAR T cells showed an enhancement of survival several weeks after treatment compared to Control mice, *n* ≥ 17 from four independent experiments **(C)**. NSG mice were subcutaneously injected with 3 × 10^5^ CD44v6^+^ MR232 tumor cells. Three days later mice were infused via tail vein injection with 4.5 × 10^6^ CD19 or CD44v6.CAR T cells and tumor growth quantified by measuring tumor size **(D)**. Mice were sacrificed at day 27, tumors were harvested and digested, cells suspension was analyzed by FACS for CD3 and CD45 (**E**, left panel), CD45RA and CD62L (**E**, middle panel), and HLA DR (**E**, right panel) expression. TILs still had cytotoxic activity on CD44v6^+^ MOLT-4 **(F)**. NSG mice were subcutaneously injected with 3 × 10^5^ CD44v6^+^ MR232 tumor cells. Three days later mice were infused via tail vein injection with 10 × 10^6^ CD19 or CD44v6.CAR T cells and tumor growth quantified by measuring tumor size **(G)**. Mice treated with CD44v6.CAR T cells showed an enhancement of survival several weeks after treatment compared to control mice *n* ≥ 13 from three independent experiments **(H)**; *p < 0.05, **p < 0.01, ***p < 0.001.

To investigate the antitumor activity of the CD44v6.CAR T cells *in vivo*, we challenged NSG mice with CD44v6^+^ human ovarian carcinoma (IGROV-1-luc) and decided to lower the number of infused therapeutic cells in order to mimic a possible dose for clinical translation (23). Mice (*n* = 4 or 5/group) were implanted with 3 × 10^5^ IGROV-1 tumor cells, 7 days later mice were infused iv with 4.5 × 10^6^ CD19 or CD44v6.CAR T cells. Tumor growth was quantified by *in vivo* imaging of luciferase expressing tumor cells and by measuring tumor size (Figures 4A,B). Several independent experiments (*n* = 4) with CAR T cells from different donors showed that a single infusion of CD44v6.CAR T cells mediates antitumor effects and significantly (*p* < 0.001) enhances survival of CD44v6 CAR T treated mice (Figure 4C).

To assess the capacity of CD44v6.CAR T to control tumor growth in a different and more aggressive tumor model, we injected NSG mice subcutaneously with the CD44v6 expressing lung adenocarcinoma cells (i.e., MR232). Three days later, mice were infused with 4.5 × 10^6^ CD19 or CD44v6.CAR T cells. Differently from what observed with IGROV-1, CD44v6.CAR T did not control tumor growth (Figure 4D). We checked if the lack of activity was due to an impaired CAR T cell recruitment and infiltration into the tumor. Eleven days after CAR T cell infusion, tumors of comparable size were harvested and processed for immunofluorescence analysis. As shown in Supplementary Figure 5, tumor cells stained with the tumor marker Cytokeratin 7, were dramatically reduced only in tumors from mice treated with CD44v6.CAR T cells, while no reduction was observed in animals treated with control cells. A huge number of human CD3^+^ cells infiltrated the tumors of CD44v6.CAR T treated mice, whereas no human CD3^+^ cells were identified in tumors from control mice. These data confirm, in the MR232 tumor model, the ability of CD44v6.CAR T cells to reach the tumor site, proliferate and kill the tumor cells (Supplementary Figure 5). CD44v6.CAR T cells were still present with an activated phenotype (Figure 4E) in the tumor at sacrifice 24 days after treatment. Moreover, after *in vitro* expansion were able to efficiently kill CD44v6-transduced MOLT-4 cells (live cells <38% at 1:1 ratio) (Figure 4F). Having excluded that the lack of *in vivo* efficacy was related to “hypofunctionality” of T cells, we speculated that, given the fast growth kinetic of MR232 tumors, the number of infused CAR T cells was not sufficient to induce regression of the established tumors. Therefore, we increased the number of infused CAR T cells to 1 × 10^7^ cells. In this setting, after an early phase in which CD44v6.CAR T treated tumors seemed to growth as CD19.CAR T treated controls, the tumors were completely controlled by CD44v6.CAR T up to day 20 (Figure 4G), when in one out of four mice, tumor started growing again. Notably, after 2 months of observation, more than 30% of CD44v6.CAR T treated mice were still alive (Figure 4H).

### CD44v6 CAR-T Cells Produced According to the GMP Manufacturing Process Retain Antitumor Activity

**Figure 5:**
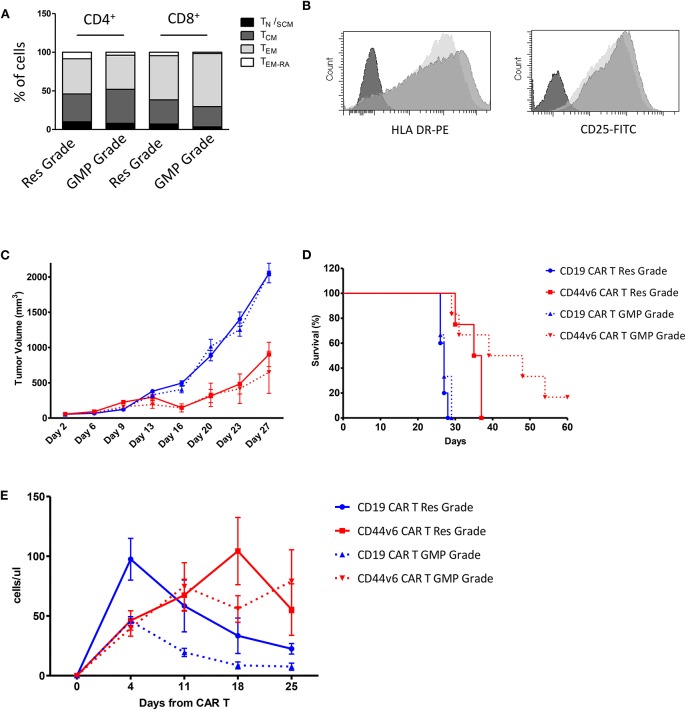
Comparison of phenotype and *in vivo* functionality of CAR T cells produced according to research or manufacturing processes. T cell memory phenotypic analysis based on CD45RA and CD62L expression. T_N/SCM_ (CD45RA^+^/CD62L^+^ memory stem T cells); T_CM_ (CD45RA^−^/CD62L^+^ central memory T cells); T_EM_ (CD45RA^−^/CD62L^−^ effector memory T cells); T_EM−RA_ (CD45RA^+^/CD62L^−^effector memory RA T cells) **(A)**. HLA DR and CD25 expression on Research Grade Product (light gray) and GMP grade Product (dark gray) **(B)**. All data are representative of two independent CAR T preparations. CAR T cells produced according to both Research Grade and GMP Grade Processes control tumor growth in mice bearing lung carcinoma **(C)** and delay mice survival **(D)**. Number of human T cells per microliter in peripheral blood of mice infused with Research Grade and GMP Grade CAR T cells at different time points **(E)**.

Given the promising results obtained with cells produced according to a method that does not meet GMP guidelines, we decided to test cells produced complying with the GMP grade CD44v6.CAR T cells manufacturing protocol, developed for the ongoing first-in-man phase I-II clinical trial in AML and MM. The major changes of the new process is the use of TransAct for T cell activation and expansion, instead of CD3/CD28 Dynal magnetic beads. T Cell TransAct is a polymeric nanomatrix conjugated to anti-CD3 and anti-CD28 mAbs. The second change in CAR T cells manufacturing is a 2-day delay for ΔLNGFR-mediated cell selection. The immunological phenotype of the CAR T cells, produced according to the two protocols is shown in Figure 5. Cells produced according to GMP grade manufacturing process, displayed the same memory phenotype (Figure 5A) and the same activation markers as compared to research grade produced cells (Figure 5B). Notably, the percentage of transduction before selection was comparable (24.5 ± 5.2 for research grade group and 25.5 ± 4.9 for GMP grade group) and expression level of CAR at the end of the process (92.3 ± 6.4 for research grade group and 95.0 ± 3.5 for GMP grade group) was comparable too (data not shown). We next verified whether the CD44v6.CAR T cells produced complying with GMP grade protocol were able to control tumor growth in the two solid tumor models studied. In the lung adenocarcinoma model, mice (*n* = 5 for the research group and *n* = 7 for the GMP group) were implanted with 3 × 10^5^ MR232 tumor cells, 3 days later mice were infused iv with 1 × 10^7^ CD19 or CD44v6.CAR T cells and tumor growth was quantified by measuring tumor size. CD44v6.CAR T cells, produced according to either method, were able to inhibit tumor growth and to extend overall survival (Figures 5C,D). Mice treated with research grade CAR T cells showed a median survival of 23 and 37 days for CD19.CAR and CD44v6.CAR T treated group, respectively (log rank test: *P* < 0.01). Similarly, mice treated with GMP grade CAR T cells showed a median survival of 21.5 and 37 days for CD19.CAR and CD44v6.CAR T treated group, respectively (log rank test: *P* < 0.05). Moreover, we assessed by FACS analysis the percentage of circulating CAR T cells at different time points, starting 4 days after CAR T cell infusion. NSG mice treated with CD44v6.CAR T cells showed a greater expansion and persistence in peripheral blood compared to control CAR T cells, and no differences were observed between Research and GMP manufacturing processes (Figure 5E). In the ovarian carcinoma model, mice (*n* = 4 for the research group and *n* = 7 for the GMP group) were implanted with 3 × 10^5^ IGROV-1 tumor cells, and seven days later were infused iv with 4.5 × 10^6^ CD19 or CD44v6 CAR T cells. As observed for lung adenocarcinoma, GMP CD44v6.CAR T cells were able to control tumor growth in a similar way as CD44v6.CAR T cells produced with research grade process (Supplementary Figure 6). Thereby, we concluded that changes introduced in the manufacturing protocol had no impact on the efficacy of the product.

## Discussion

In this study, we investigate the antitumor activity of CD44v6-specific CAR T cells in xenogenic models of solid tumors. In agreement with published studies on primary and metastatic tumors (9), we found that CD44v6 is broadly expressed in most cell lines from solid tumors of various histotypes, including ovarian and lung carcinomas, which were used to perform the current study. It is known that expression level of the target antigen may affect CAR T cell functionality and persistence (24). Therefore, to challenge the effectiveness of the CAR T cells in a more real model, we selected tumor cell lines (i.e., IGROV-1 and MR232) expressing moderate level of CD44v6.

Moreover, to overcome the CAR T cell product variability related to the different transduction efficiency across individuals, we took advantage of the human low affinity Nerve Growth Factor receptor (ΔLNGFR)-spacer present in the CD44v6.CAR (13). The ΔLNGFR enables to efficiently enrich CAR T cells with a validated clinical-grade technology (12, 13), thus allowing the manufacturing of more standardized CAR T cell products with narrowed variability.

The impact that the variable T cell subset composition of CAR T cell products have on clinical outcome are currently under study to identify the more effective T cell subset in term of efficacy and safety (25). The CD4 and CD8 composition of the CD44v6.CAR T cell products was quite variable with a prevalence of CD4 T cells. Based on our experience on RVV-mediated T cell transduction (12, 26), this variability is independent of the manufacturing process and strongly related to intrinsic characteristic of the patients/donors. It is still a pending issue what is the ideal CD4 and CD8 composition. As an example, it was demonstrated that in neuroblastoma patients, antitumor activity and long-term fate of infused T cells is highly concordant with the percentage of CD4^+^ cells (27). Moreover, in a phase I trial of children and young adult with relapsed or refractory B-lineage ALL, Gardner and colleagues, using a CAR T cell products with a defined CD4/CD8 composition, observed a very impressive remission rates and prolonged overall survival (28). Anyway, at present it is not possible to predict if CD4/CD8 CAR T cells composition could be correlated with long-term survival.

It is well known that clinical responses often correlate with CAR T cell expansion and persistence (1, 2). Moreover, memory phenotype directly dictates the self-maintenance capacity of tumor specific T cells (29). Indeed, it has been demonstrated that human CD19.CAR T cells manufactured from central memory or naïve T cells are more potent than CD19.CAR T cells manufactured from effector memory T cells or from circulating PBMC, in the elimination of CD19^+^ tumors in immunodeficient mice (30).

The current CAR T cell manufacturing processes require *ex vivo* activation and expansion of the patient's T cells. This may speed up effector T cell differentiation and functional exhaustion, with a negative impact on the T cell phenotype, thereby reducing the potency of the CAR T product (31). Therefore, we verified whether CD44v6.CAR T cells, generated from PBMC in the presence of homeostatic cytokines IL-7 and IL-15, maintained a functional phenotype and their potency. Since T cell activation can promote a reprogramming of T cell functions and in the worst cases can induce exhaustion (32, 33), we verified whether CD44v6.CAR T maintained their potency. Indeed, CD44v6.CAR T partially retained an early-differentiated memory phenotype at the end of the manufacturing process and were able to degranulate, produce cytokines and mediate cytotoxicity even at low effector to target ratio, when exposed to CD44v6^+^ tumor cells but not to CD44v6^−^ target cells.

In ovarian and lung carcinoma-bearing mice, adoptive transfer of CD44v6.CAR T cells resulted in a high degree of T cell infiltration in the tumors, probably due to CD44v6.CAR T proliferation, as demonstrated by Ki67 staining of tumor infiltrating cells.

Since several studies reported low CAR T cell expansion in the treatment of solid tumor, we wondered which features lead to CD44v6.CAR T cells accumulation in our setting. First, we hypothesized a role for the IL 7 and IL15 cytokines used during the manufacturing process that allowed the generation of T cells endowed with an early differentiation phenotype with no evidence of cell exhaustion. In agreement, serial antigen stimulations did not affect CD44v6.CAR T proliferation and functions, thus supporting the observed *in vivo* expansion and antitumor activity.

Moreover, it was demonstrated that persistence of adoptively transferred T cells is influenced by all the components (i.e., costimulatory domains, spacers and linkers) of the CAR structure (34). In particular, costimulation can impact CAR T cell persistence and exhaustion, being usually the 4-1BB-bearing CAR T cells more persistent than CD28-bearing CAR T cells (34). There are, however, exceptions related to the experimental setting; CD44v6.CAR T cells endowed with 4-1BBζ costimulatory endodomain displayed lower antileukemic effect with more aggressive cytokine release syndrome compared to CD44v6.CAR T cells with the CD28/ζ construct (35). No differences between the two costimulatory endodomains were observed in CD19.CAR T cells (35). The structural features influencing CD44v6.CAR T cell persistence and proliferation are a major focus of our ongoing studies.

The therapeutic activity of CAR T can be affected by the selection of antigen-loss variants (2), in particular when the targeted antigen shows heterogeneous expression within the tumor mass, as often happens in solid tumors. In our xenogeneic tumor models, the lack of complete tumor eradication was not due to the selection of CD44v6 negative tumor cells. Indeed, CD44v6 expression on tumor cells harvested at the endpoint from mice treated with CD44v6.CAR or control CAR T cells were comparable.

The observation that in most treated mice, albeit delayed, the tumors progress, suggests the action of inhibitory mechanisms that remain to be explored. For instance, we know that MR323 express PD-L1 an immune checkpoint receptor ligand, which may lead to T cell exhaustion and tumor escape from immunosurveillance (36). Moreover, the use of NSG mice deficient in adaptive and innate immunity (37), could limit the generation of fully effective antitumor immune responses due to the absence of a supportive environment able to produce cytokines to sustain CAR T cells (38) and to generate endogenous antitumor effectors via epitope spreading, a mechanism crucial in controlling tumor growth (39). A more informative analysis of the antitumor potential of CD44v6.CAR T cells would rely on the use of immunocompetent tumor models.

Manufacturing of CAR T cells for clinical trials needs to occur according to rigorous rules that are defined under GMP guidelines. Since the ultimate focus of our research is the clinical translation, we investigated whether CD44v6.CAR T cells produced with clinically-complainant procedures maintained the same antitumor activity observed with the research grade process. Our studies showed that manufacturing of GMP-grade CD44v6.CAR T cells does not affect CAR T cells phenotype in term of differentiation and activation, features that have a strong impact on antitumor therapeutic activity (33). Moreover, we demonstrated that GMP-grade CD44v6.CAR T cells are endowed with antitumor activity and extend overall survival in two solid tumor models.

In conclusion, these results support the clinical application of CD44v6.CAR T adoptive cell therapy for the treatments of solid tumors.

## Data Availability Statement

All data generated and analyzed for this study are included in the article/Supplementary Material.

## Ethics Statement

The animal study was reviewed and approved by IACUC: Institutional Animal Care and Use Committee of the San Raffaele Hospital.

## Author Contributions

SP and CA designed and conducted laboratory experiments, analyzed data, and wrote the manuscript. SC, EC, VV, AS, and BV conducted laboratory experiments. CB and BV revised the manuscript. CT designed and supervised the study, revised the manuscript, and acted as senior author.

### Conflict of Interest

SP, CA, SC, EC, VV, AS, BV, and CT are employees of MolMed SpA, the company is the applicant of patents on CAR molecules containing LNGFR derived spacers including the specific CD44v6 targeted CAR studied in this work and on the HSV-TK Mut2 suicide gene. CB is chairman of Molmed Scientific Advisory Board.
