# Supplementary figures and images for: CAR T Cells Redirected to CD44v6 Control Tumor Growth in Lung and Ovary Adenocarcinoma Bearing Mice
Source: Front Immunol. 2020 Feb 4;11:99. doi: 10.3389/fimmu.2020.00099 (PMC7010926; doi:10.3389/fimmu.2020.00099)

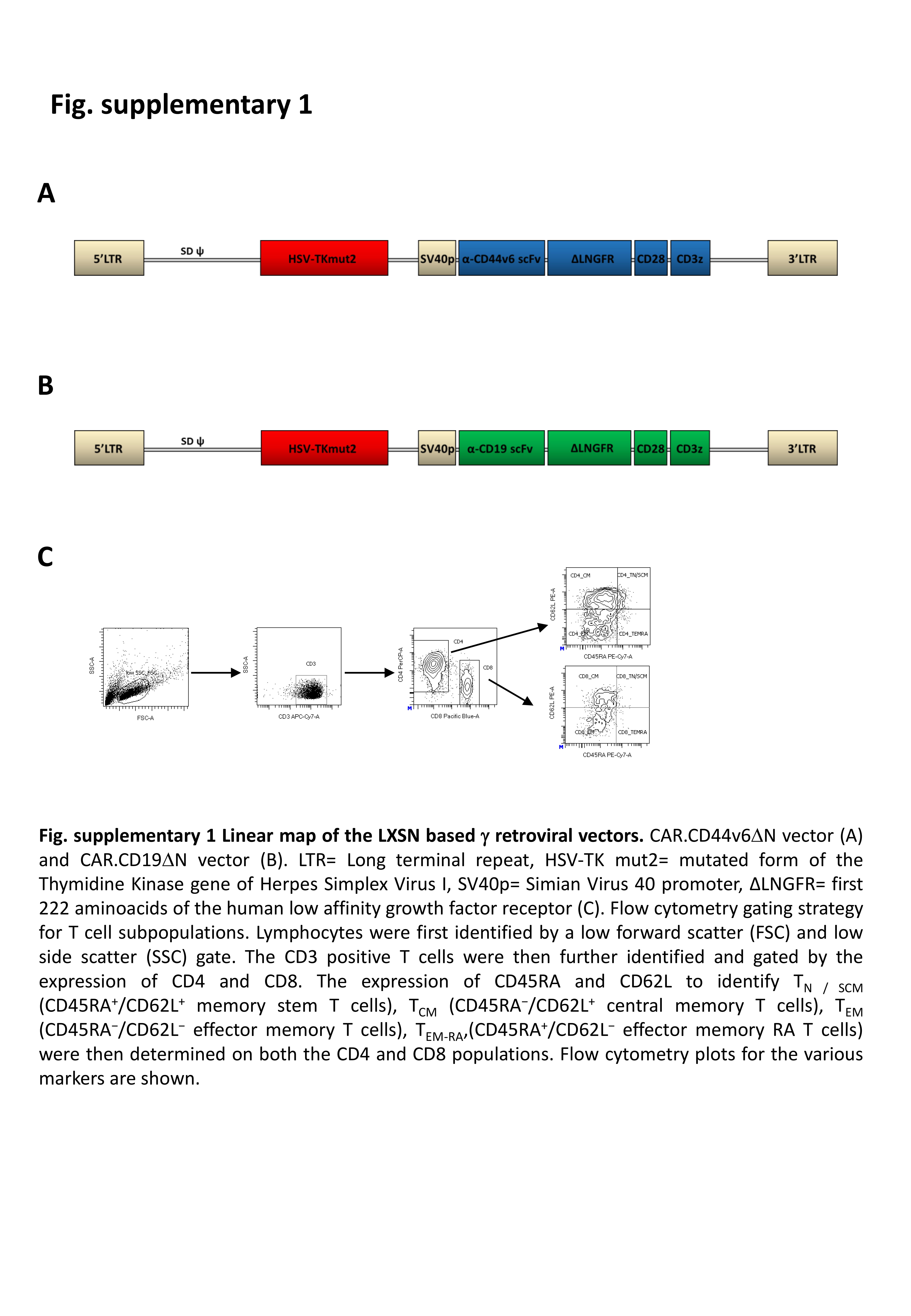

Supplement: Supplementary file 1 [file Image_1.TIF]

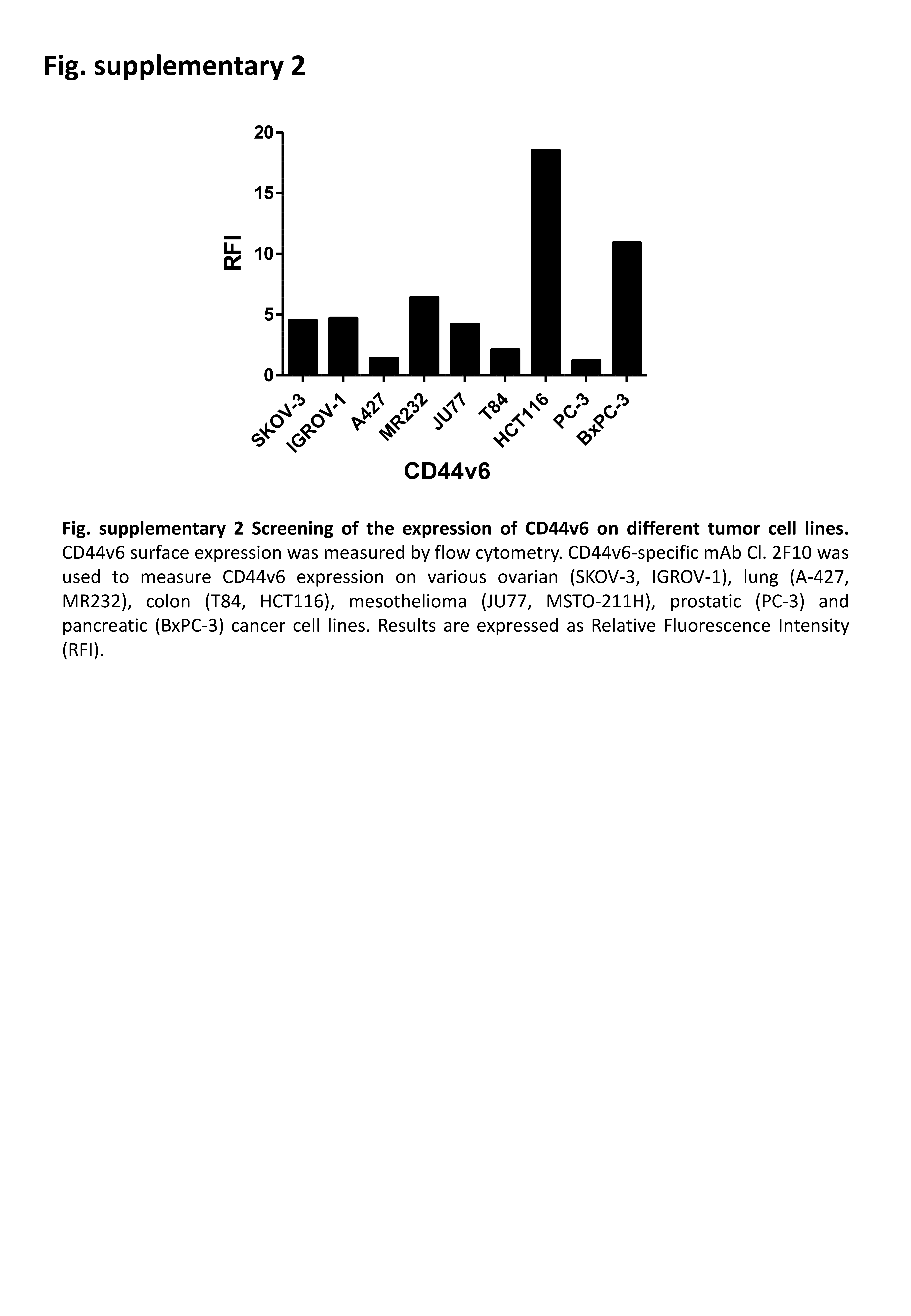

Supplement: Supplementary file 2 [file Image_2.TIF]

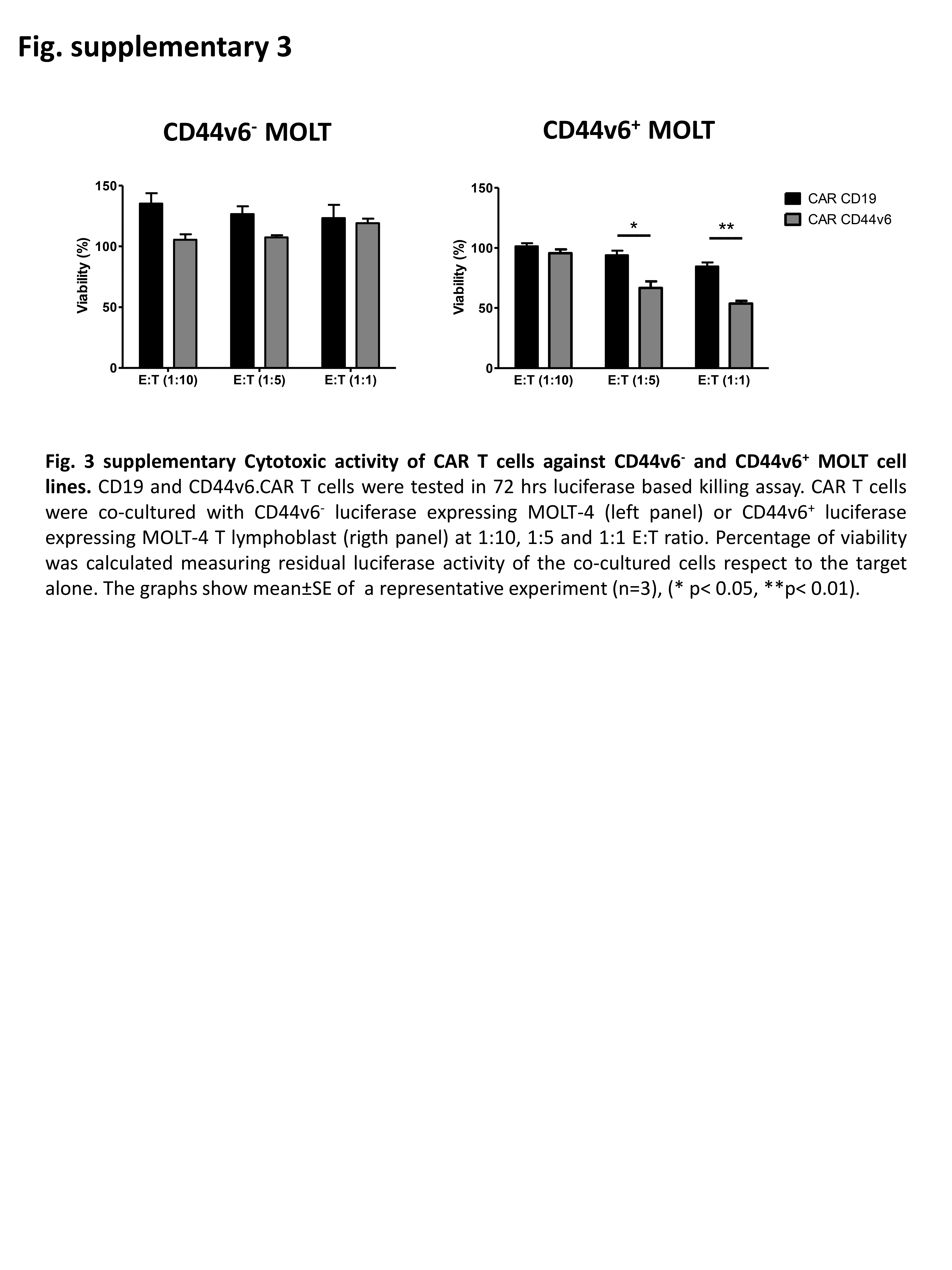

Supplement: Supplementary file 3 [file Image_3.TIF]

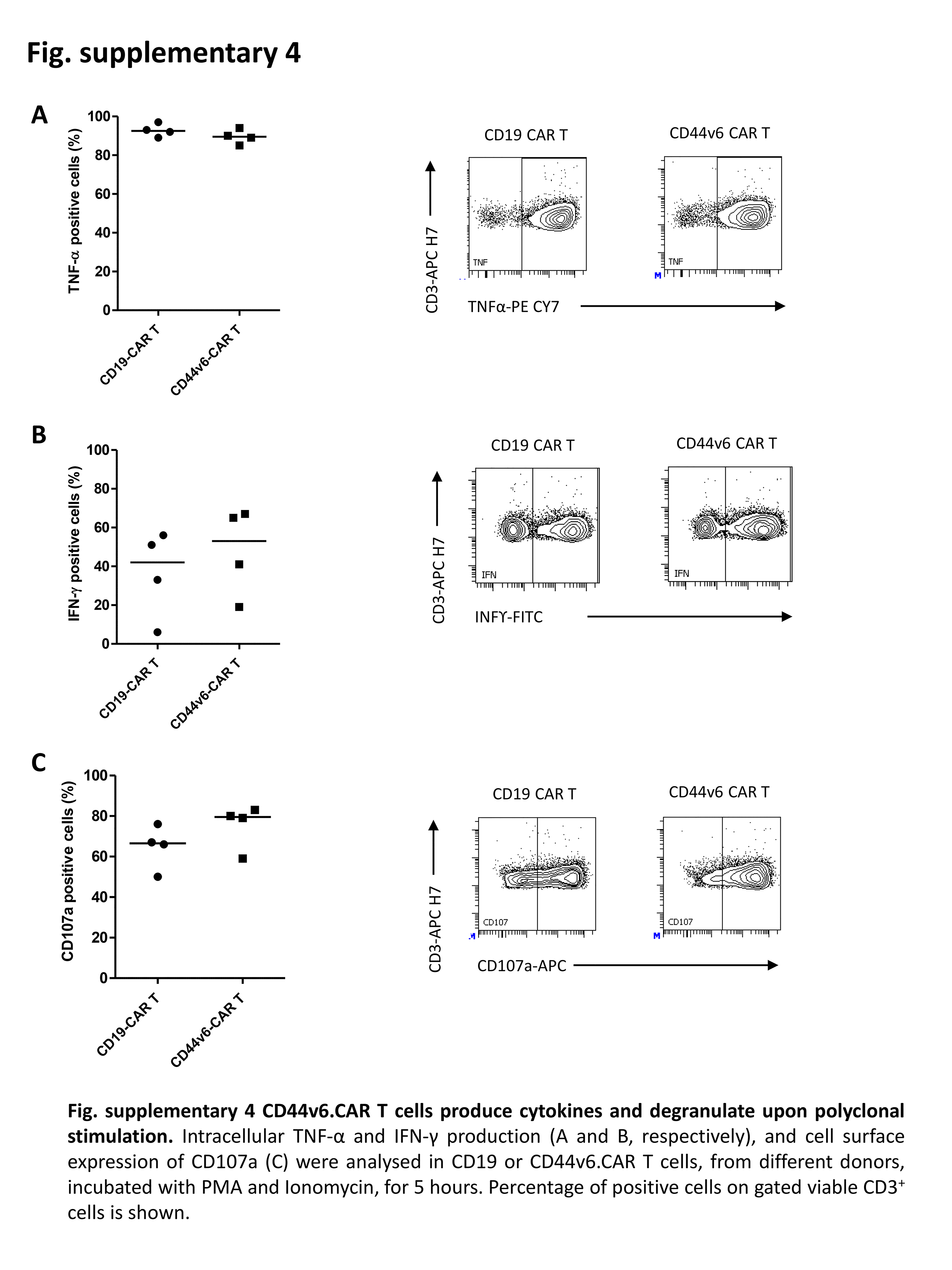

Supplement: Supplementary file 4 [file Image_4.TIF]

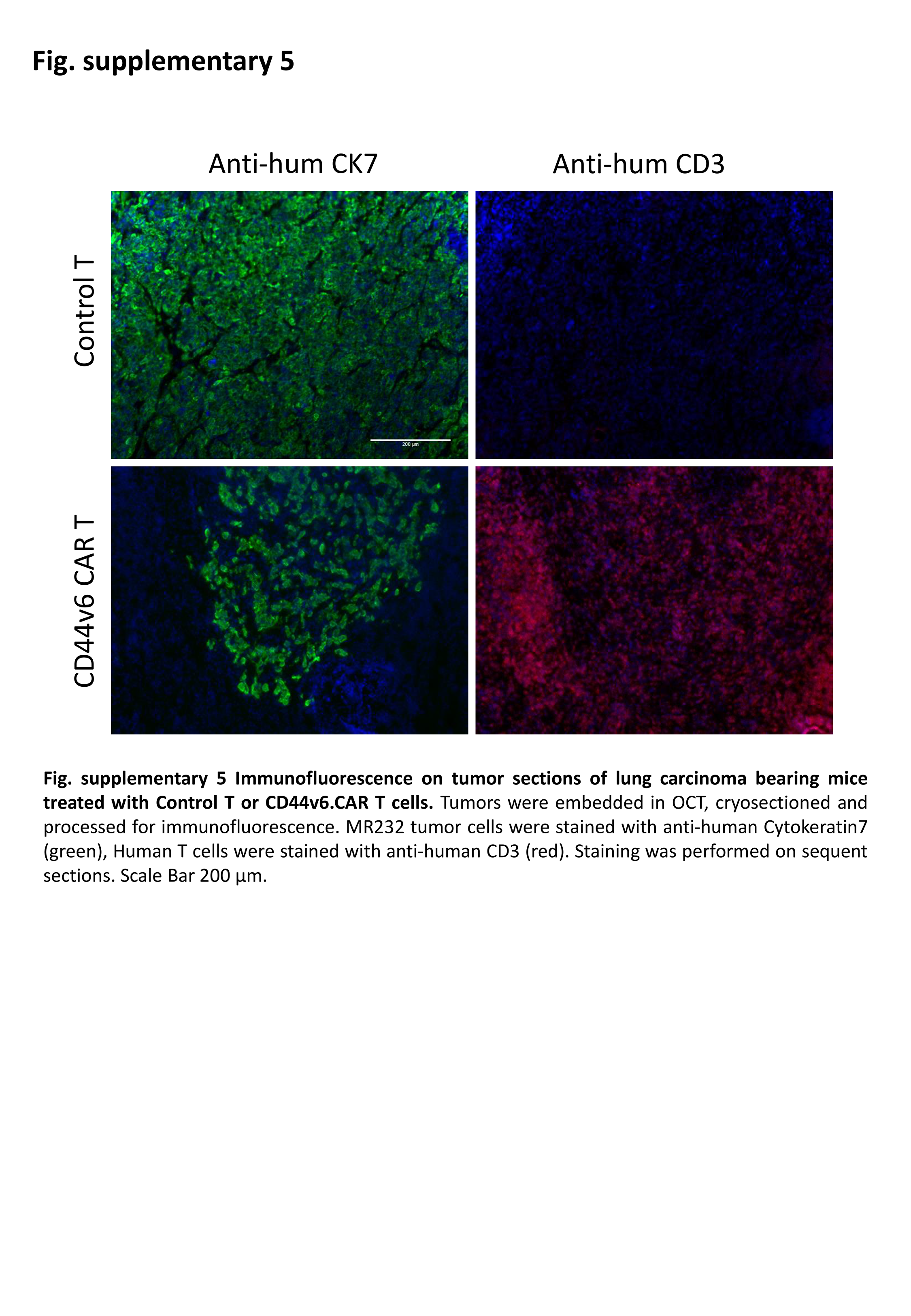

Supplement: Supplementary file 5 [file Image_5.TIF]

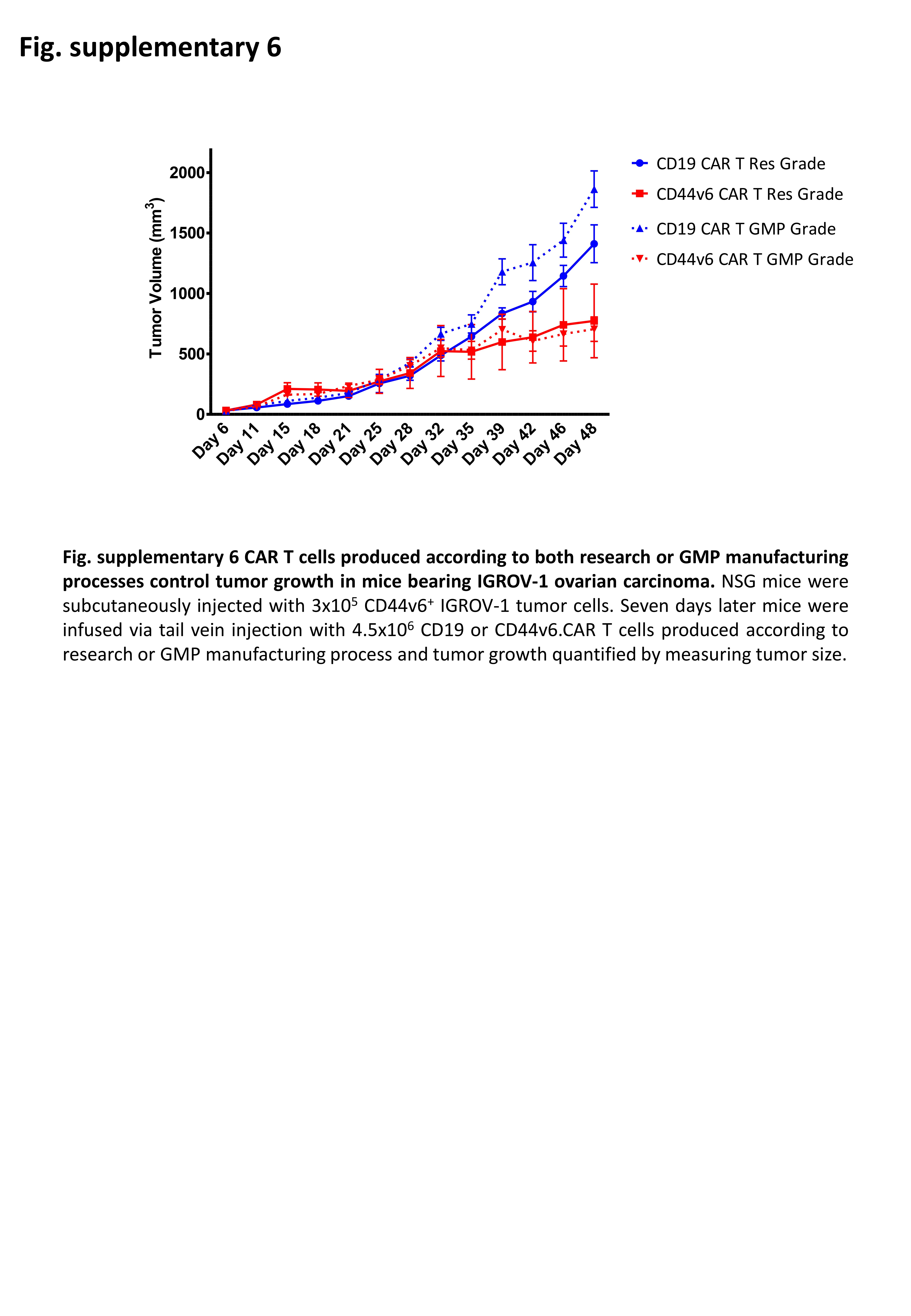

Supplement: Supplementary file 6 [file Image_6.TIF]
